# Reconstruction of Elbow by Free Fibular Graft in a Case of Osteoclastoma of Proximal Ulna: A Rare Case Report

**DOI:** 10.1155/2015/429309

**Published:** 2015-08-12

**Authors:** Kiran Kalaiah, S. G. Thejaswi, Marula Siddappa

**Affiliations:** ^1^Department of Orthopedics, Mysore Medical College and Research Institute, Irvin Road, Mysore, Karnataka 570001, India; ^2^Department of Orthopedics, Sikkim Manipal Institute of Medical Science, 5th Mile Tadong, Gangtok, Sikkim 737101, India

## Abstract

Giant cell tumour is a benign aggressive bone tumour. Most commonly, it is seen in epiphysiometaphyseal region around knee and distal radius. Proximal ulna is a rare location for giant cell tumour. According to reports, only 4 such cases have been reported in English literature. We report one such case of giant cell tumour of proximal ulna. Patient presented with painless, progressive swelling around right elbow since 4 months. Proximal ulna along with tumour was resected and elbow was reconstructed using nonvascularized free fibular graft. At two years of follow-up, patient is tumour-free and has functional range of movement in elbow. We are reporting the case because of its rare location and for the indigenous treatment modality of using free fibular graft for elbow reconstruction.

## 1. Introduction

Giant cell tumour also known as osteoclastoma is a benign aggressive tumour. It has a slight female preponderance and is seen in the age group of 20–40 years. Giant cell tumour is an epiphysiometaphysial tumour commonly seen in distal end of femur, proximal end of tibia, and distal end of radius [1].

Treatment options include curettage with or without bone grafting, extended curettage, wide excision, and reconstruction.

We are reporting a case of giant cell tumour of proximal ulna. According to reports, only few such cases have been reported in English literature. After evaluation, tumour was resected en bloc and joint was reconstructed using free fibular graft. This case is being reported for its rare location and the indigenous treatment modality carried out.

## 2. Case History

**Figure 1:**
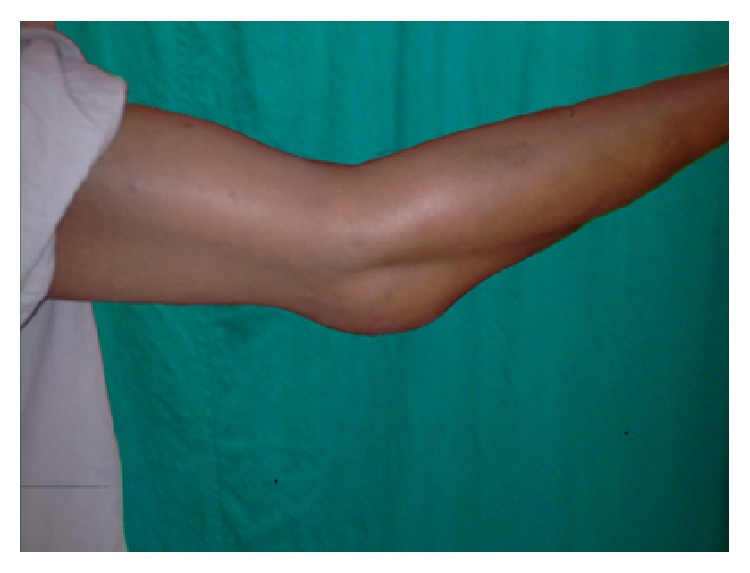
Swelling at the time of presentation.

A 30-year-old male presented with painless swelling around his right elbow since 4 months (Figure 1). It was insidious in onset with gradual increase in size which later on started to affect his elbow movements.

On examination, a localised swelling measuring 5 × 6 cm in posterior aspect of elbow was seen which was nontender, hard in consistency, and continuous with proximal ulna. The overlying skin was normal. Elbow flexion was up to 90 degrees and terminal 10 degrees of extension were limited.

**Figure 2:**
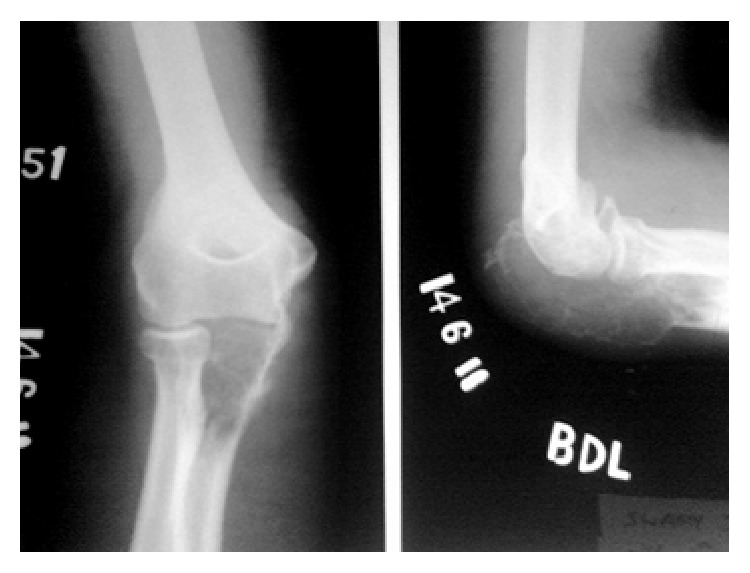
X-ray at the time of presentation.

X-ray showed a lytic lesion involving entire proximal ulna with soap bubble appearance with destruction of articular surface (Figure 2). FNAC was done to aid the diagnosis and it showed features suggestive of giant cell tumour.

Giving a tumour-free upper limb along with a functional elbow joint was a challenging part. Hence, treatment plan included en bloc excision of proximal ulna with tumour and safe margin of normal appearing bone and reconstruction of the elbow joint using free fibular graft.

### 2.1. Surgical Procedure

**Figure 3:**
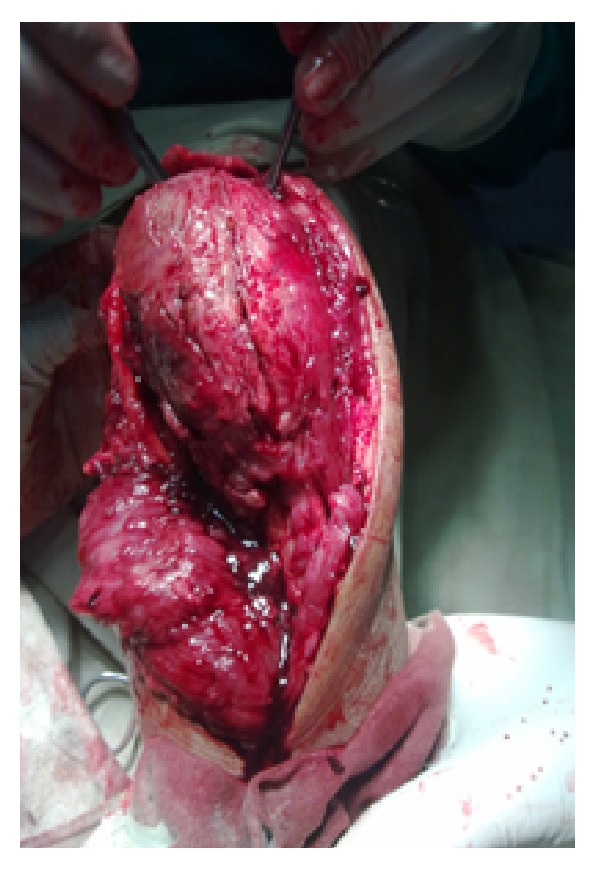
Peroperative picture of tumour.

**Figure 4:**
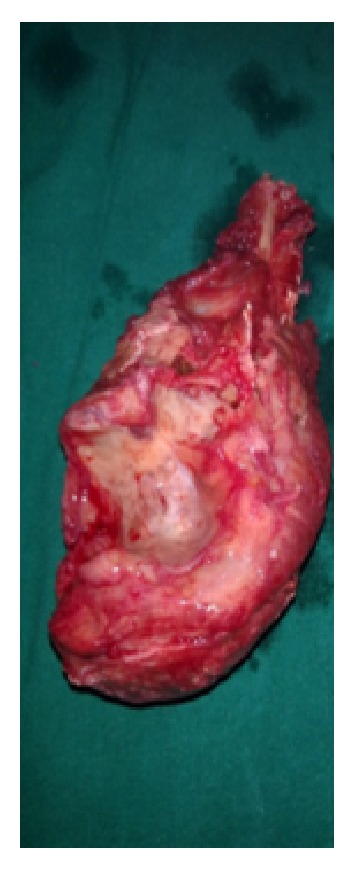
Excised tumour.

**Figure 5:**
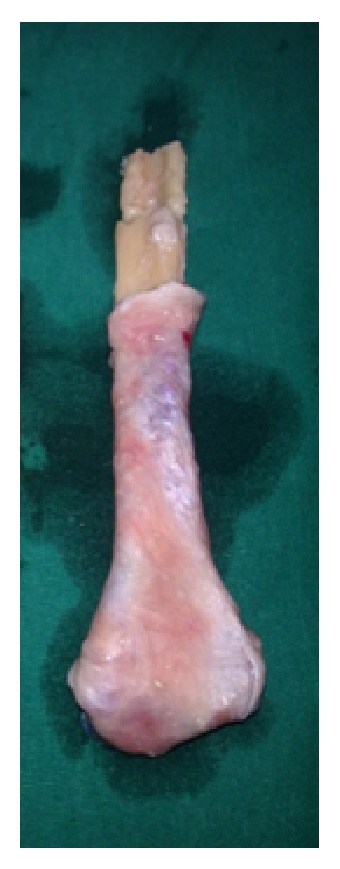
Prepared graft.

**Figure 6:**
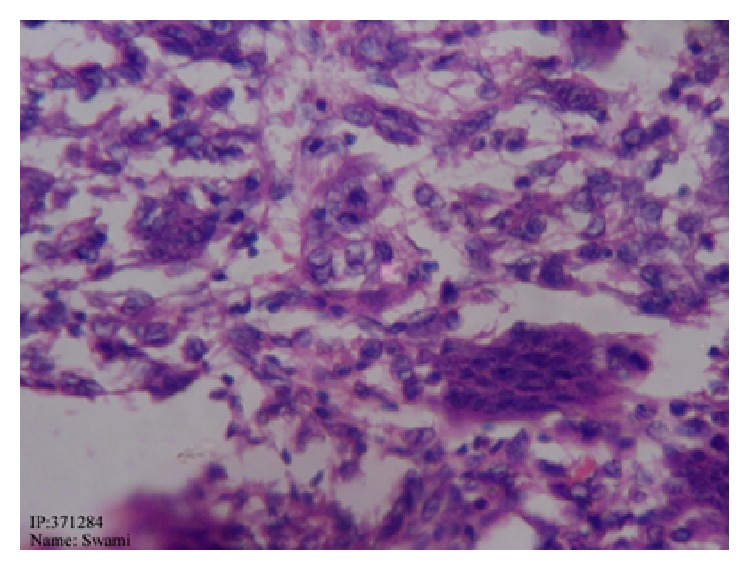
Histopathology showing giant cell in cluster.

Under general anaesthesia, tumour was exposed through posterior approach to elbow (Figure 3). Proximal ulna with tumour and 2 cm of normal bone was excised (Figure 4). Another team of surgeons harvested the required length of free fibular graft from upper fibula from ipsilateral leg. Head of the fibula was shaped to match the olecranon process of ulna and tensor fascia lata was covered over fibular head to act as articular surface (Figure 5). Graft was fixed to ulna using semitubular plate and a K-wire was put for temporary immobilisation of elbow in 90-degree flexion. Triceps tendon was sutured to the graft at proximal end. Specimen was sent for histopathological examination which confirmed the diagnosis of giant cell tumour (Figure 6). Immediate post-op X-ray confirmed the proper fixation.

### 2.2. Follow-Up

Post-op period was uneventful. K-wire was removed after 3 weeks and elbow was mobilised. Patient was regularly followed up for 2 years for radiological union of the graft, functional outcome of the elbow, and possible recurrence or metastasis to lungs.

**Figure 7:**
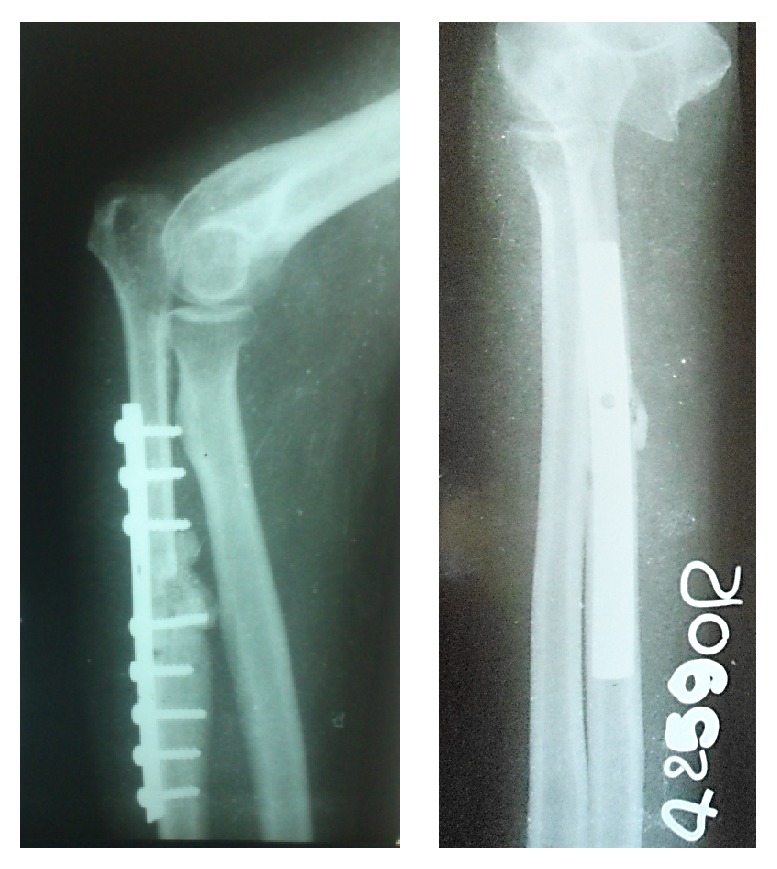
Follow-up X-ray after 2 years.

Radiological union of the graft was seen as early as 4 months. At the end of 2 years, patient is pain-free and has a stable and functional elbow (Figure 7). No recurrence or metastasis of tumour was seen. Donor site is also pain-free and patient is walking comfortably.

### 2.3. Discussion

Giant cell tumour is a benign bone tumour but has an aggressive course. It is seen after skeletal maturity with slight female preponderance. Epiphysiometaphysical region around knee and distal end radius are the favoured sites [1]. Very few cases of giant cell tumour affecting proximal ulna have been reported.

First case of giant cell tumour of proximal ulna in South East Asia was reported by Sanjay et al. in 1991 [2]. And since then, only one case has been reported in this region. To the best of our knowledge, this is only the third that is being reported from India and the 5th case ever being reported in English literature [2, 3].

Usual management of this tumour involves resection of the tumour or curettage and ablation of the cavity surface. This is feasible if the tumour is located in a long bone. In a small bone, excision and reconstruction are preferred. The challenge we faced was that the tumour involved proximal part of ulna along with elbow joint of the dominant upper limb of the patient. Hence, it was necessary for us to keep the bone and elbow joint-free from tumour and give the patient a stable, functional elbow joint. In previous reported cases, excision of proximal ulna along with arthrodesis of elbow in functional position has been done [2, 3]. We planned to excise the entire proximal ulna with tumour and 2 cm of visible normal bone and reconstruction of elbow joint using proximal free fibular graft along with tensor fascia lata coverage to act as articular surface.

Free fibular grafts have been used frequently in orthopaedics as a graft to bridge long bony gaps and in very few cases to reconstruct joints. There have been reports of them being used to reconstruct elbow joint after resection of distal end of humerus [4–6]. But reconstruction of ulnar component of elbow has rarely been attempted. Usui et al. reported that the use of a free vascularized fibular graft including the head carried a risk of fibular head collapse [7]. But such a thing has not been noticed yet by us till now in this case.

In summary, we were able to perform a limb saving procedure along with giving a stable functional joint using a free fibular graft. However, further follow-up and more research are needed in this regard to carry out this procedure as a regular treatment modality for tumours around elbow.
